# Moderate-intensity continuous training reduces triglyceridemia and improves oxygen consumption in dyslipidemic apoCIII transgenic mice

**DOI:** 10.1590/1414-431X2024e13202

**Published:** 2024-07-29

**Authors:** N.R. Teixeira, D.R. Jimenes, C. Schultz, D.L. Almeida, P.C.F. Mathias, J.A. Berti

**Affiliations:** 1 Departamento de Ciências Fisiológicas, Universidade Estadual de Maringá, Maringá, PR, Brasil; 2 Departamento de Educação Física, Universidade Estadual de Maringá, Maringá, PR, Brasil; 3 Departamento de Biociências e Fisiopatologia, Centro de Ciências da Saúde, Universidade Estadual de Maringá, Maringá, PR, Brasil; 4 Laboratório Experimental em DOHaD, Departamento de Biotecnologia, Genética e Biologia Celular, Universidade Estadual de Maringá, Maringá, PR, Brasil

**Keywords:** Lipid metabolism, Dyslipidemia, Physical exercise, ApoCIII, Oxygen consumption

## Abstract

This study aimed to investigate metabolism modulation and dyslipidemia in genetic dyslipidemic mice through physical exercise. Thirty-four male C57Bl/6 mice aged 15 months were divided into non-transgenic (NTG) and transgenic overexpressing apoCIII (CIII) groups. After treadmill adaptation, the trained groups (NTG Ex and CIII Ex) underwent an effort test to determine running performance and assess oxygen consumption (V̇O_2_), before and after the training protocol. The exercised groups went through an 8-week moderate-intensity continuous training (MICT) program, consisting of 40 min of treadmill running at 60% of the peak velocity achieved in the test, three times per week. At the end of the training, animals were euthanized, and tissue samples were collected for *ex vivo* analysis. ApoCIII overexpression led to hypertriglyceridemia (P<0.0001) and higher concentrations of total plasma cholesterol (P<0.05), low-density lipoprotein (LDL) cholesterol (P<0.01), and very low-density lipoprotein (VLDL) cholesterol (P<0.0001) in the animals. Furthermore, the transgenic mice exhibited increased adipose mass (P<0.05) and higher V̇O_2peak_ compared to their NTG controls (P<0.0001). Following the exercise protocol, MICT decreased triglyceridemia and cholesterol levels in dyslipidemic animals (P<0.05), and reduced adipocyte size (P<0.05), increased muscular glycogen (P<0.001), and improved V̇O_2_ in all trained animals (P<0.0001). These findings contribute to our understanding of the effects of moderate and continuous exercise training, a feasible non-pharmacological intervention, on the metabolic profile of genetically dyslipidemic subjects.

## Introduction

Hypertriglyceridemia disrupts metabolism and boosts the development of metabolic diseases such as obesity, diabetes, and non-alcoholic fatty liver disease (1-3). Furthermore, it is strongly associated with atherosclerosis and cardiovascular diseases (4,5). The metabolism of triglycerides (TG) and cholesterol (CHOL) is dynamic and complex, and hypertriglyceridemia etiology is multifactorial, including environmental and genetic factors (6).

Synthesized mainly in the liver, elevated levels of apolipoprotein CIII (apoCIII) is commonly detected in hypertriglyceridemia, hypercholesterolemia, and increased free fatty acids (7,8). ApoCIII is a key apolipoprotein found in the triglyceride-rich lipoproteins [very low-density lipoprotein (VLDL), intermediate-density lipoprotein (IDL), low-density lipoprotein (LDL), and high-density lipoprotein (HDL)]. A consolidated rodent model for the study of hypertriglyceridemia is the C57Bl/6 transgenic mice overexpressing human apoCIII (9). In them, the overexpression of apoCIII slows down the removal of TG from lipoproteins, decreasing their affinity for lipoprotein lipase (LPL) and reducing the uptake of VLDL and chylomicrons via the hepatic receptor (10-12), leading to hypertriglyceridemia in the animals.

Among different approaches aimed at reducing the damage of dyslipidemia and other metabolism-related illnesses, physical exercise is a non-pharmacological intervention often used with a good level of efficiency and safety (13). Moderate-intensity continuous training (MICT) is a physical exercise with a high rate of lipid oxidation for energy substrate, both during and after exercise (14). Furthermore, aerobic physical training in rodents has been shown to attenuate deleterious effects of a high-fat diet (15) and developmental chronic obesity (16).

Although physical exercise has been widely documented in the literature as effective in fighting dyslipidemia in humans and in animal models, much remains to be elucidated regarding the modulation of dyslipidemia induced by genetic alterations through physical exercise. Therefore, this study aimed to investigate the effect of 8 weeks of MICT on lipid and glycolytic metabolism and V̇O_2_ in transgenic mice expressing human apoCIII.

## Material and Methods

Non-transgenic (NTG) mice were bred with apoCIII-tg mice in accordance with international guidelines, and their offspring were used in this study. NTG and apoCIII-tg mice donated by Dr. Helena Coutinho Franco de Oliveira from the State University of Campinas (UNICAMP) were housed in the Animal Facility of the Department of Physiological Sciences of State University of Maringá (DFS-UEM), with controlled 12-h light-dark cycle and room temperature (23±1°C), in a ventilated rack with *ad libitum* access to water and commercial chow (Nuvilab^®^, Brazil). All the protocols used in the present study were in accordance with national and international (ARRIVE) guidelines and were approved by the Biosafety Committee (CTNBio No. 819/2013) and the Committee of Ethics in the Use of Animals in Experimentation (CEUA No. 020/2013) of the State University of Maringá.

Genotyping was confirmed by measuring basal triglyceride levels after a 12-h overnight fast. Animals with triglyceride levels lower than 100 mg/dL were considered NTG and animals with triglyceride levels higher than 300 mg/dL were considered transgenic (CIII) (17-19).

For the experiments, 34 male mice with an average age of 15 months were individually housed, being 18 CIII mice and 16 NTG mice. Ten CIII mice and eight NTG mice underwent the aerobic training protocol (NTG Ex and CIII Ex), and the remaining 8 mice from each group formed the sedentary control groups (NTG Sed and CIII Sed).

### Treadmill adaptation

The adaptation period consisted of one week of training with a light and fixed load (16 cm/s) and progressively increasing duration, starting with 10 min on the first day and ending with 20 min. After the adaptation period, the animals had 24 h of rest without any physical exertion or stress before undergoing the first maximal exercise test.

### Maximum effort test

The maximum effort test began with a 5-min warm-up at 10 cm/s, followed by an increase in load of 9 cm/s every 3 min until the animal reached exhaustion (20). The effort test was conducted at two time points: before the training to assess the animals' baseline conditioning and after the training protocol to evaluate the physiological responses after 8 weeks.

The tests were performed on an individual treadmill designed for rodents, connected to a gas analyzer to measure V̇O_2_ during the run (Panlab Harvard Apparatus^®^, USA). We considered the animal to have reached exhaustion when it could no longer keep up with the running pace and had given up running at least 3 consecutive times, keeping only the front limbs on the platform.

Based on the results of the first effort test, the training intensity was prescribed using the peak velocity (V_peak_) reached during the test. For a moderate intensity level, we used 60% of the animals' V_peak_. Since there was no significant difference in the animals' performance in the effort test, the established velocity for the trained groups was 35 cm/s.

### MICT protocol

The MICT protocol consisted of alternate-day training sessions, three times a week, with each session lasting 44 min: 2 min of warm-up at 16 cm/s, 40 min of training at 35 cm/s, and 2 min of cool-down at the same intensity as the warm-up (21). The behavior of the animals during the run was recorded as good or insufficient, and the animals that rejected the training were identified. Animals that had given up running in five or more sessions, representing ≥20% of the training, were excluded from the analysis.

### Euthanasia, blood, and tissue collection

At the end of the training, the animals underwent an effort test, as described. Following 24 h of rest from the last effort test, the animals were anesthetized with isoflurane (Isoforine^®^, Cristália, Brazil) and euthanized. For the experiments, exsanguination was first performed to obtain plasma samples, followed by laparotomy to collect total adipose tissue, liver, and muscles (gastrocnemius and soleus).

### Plasma biochemical assays

Caudal blood samples were collected to assess glucose levels using a glucometer (ACCU-CHEK^®^, Roche Diabetes Care, Switzerland). For TG and cholesterol, we used commercial kits (Labtest^®^; Gold Analisa, Brazil). Retro-orbital blood samples were collected to evaluate HDL cholesterol and LDL cholesterol using commercial kits (Labtest^®^; Gold Analisa). VLDL cholesterol was calculated using the Friedewald equation (VLDL=TG/5).

### Muscle glycogen content

Approximately 1 g of muscle tissue from the soleus and gastrocnemius muscles of both legs were collected and macerated in liquid nitrogen with perchloric acid (0.6 N). The resulting mass was homogenized and centrifuged (10 min at 2815 *g*, 4°C), and a fraction of 100 µL of the supernatant was used to determine the levels of free glucose (22).

The other fraction of the supernatant (100 µL) was mixed with amyloglucosidase (50 µL), bicarbonate (50 µL), and sodium acetate (960 µL). The solution was incubated at 40^o^C in a water bath with agitation for 2 h, and the enzymatic reaction was stopped by adding perchloric acid (0.6 N, 500 µL). After another centrifugation (10 min, ∼280 *g*, 4°C) , the supernatant (100 µL) was used to determine the total concentration of free glucose, including glucose derived from glycogen (23).

### Liver fat extraction

Approximately 1 g of liver tissue was homogenized with a solution containing equal concentrations of chloroform and methanol. Subsequently, this solution was filtered, centrifuged for 10 min (50 *g*, 27°C), and stored in an oven at 40°C for 72 h. The weight of the resulting fat was recorded every 24 h. After 72 h in the oven, the residual fat was weighed using a precision scale (Shimadzu^®^, ATY 224R, Japan).

### Adipocyte histology

The perigonadal adipose tissue was first dehydrated in xylene glycol and then embedded in paraffin. For slide preparations, the material was sectioned into semi-serial sections of 5 µm thickness using a microtome (Leica RM 2145^®^, Japan) and stained with hematoxylin-eosin. Adipocyte morphometry was performed by measuring 100 adipocytes per animal captured using a 20× objective lens with the aid of a microscope (MOTIC^®^, China) and the Image-Pro Plus^®^ software (Media Cybernetics Inc., USA).

### Statistical analysis

The data were processed using the statistical software GraphPad Prism^®^ (version 9.0, USA). We assessed the normality of the data using the Shapiro-Wilk test. For normally distributed data, we performed two-way ANOVA, followed by *post hoc* Tukey's test. The data are reported as means±SD with a significance level set at P<0.05.

## Results

### Body mass, water and food consumption, and organs

**Figure 1:**
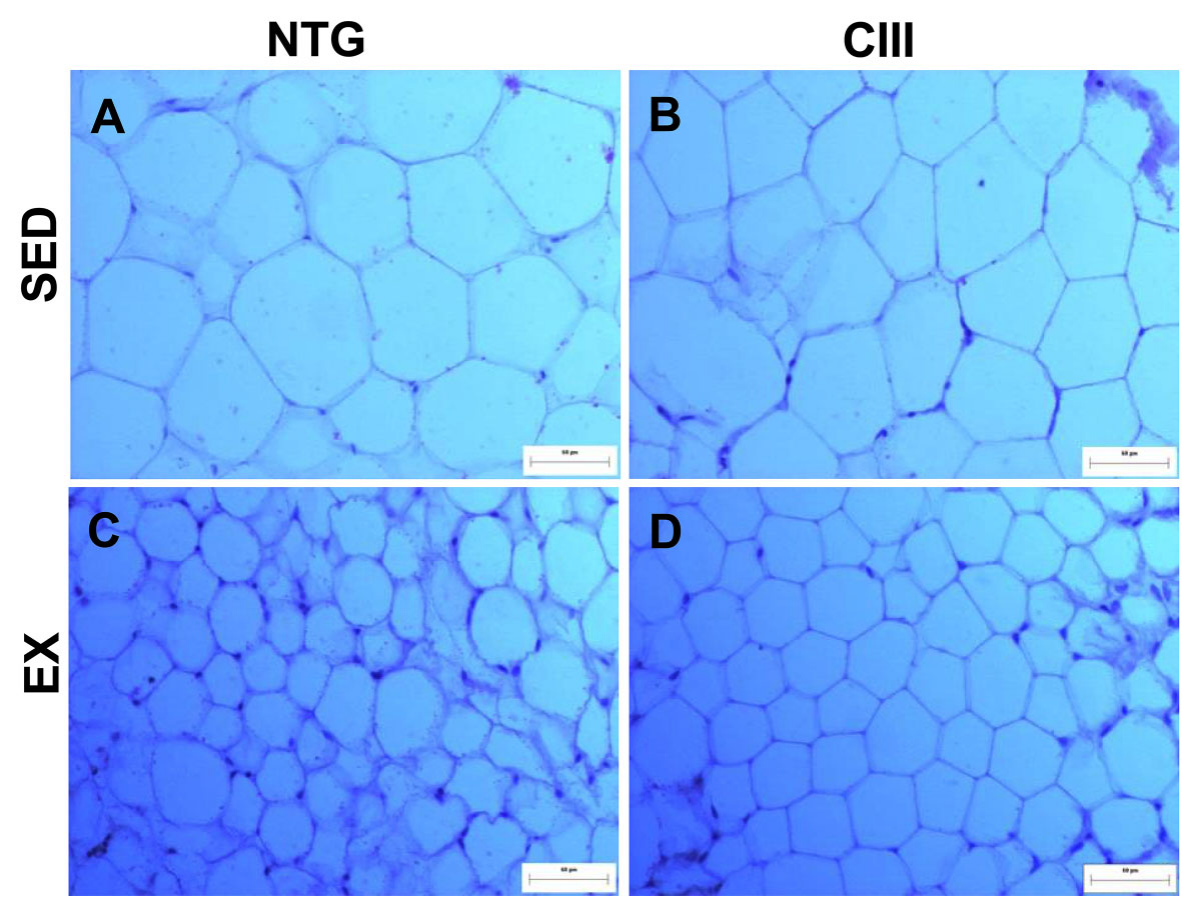
Histology of the perigonadal adipose tissue stained with the hematoxylin/eosin technique. Photomicrographs captured with an optical microscope using a 20× objective lens (scale bar 80 µm^2^). **A**, Non-transgenic sedentary (NGT Sed) group; **B**, transgenic overexpressing apoCIII sedentary (CIII Sed) group; **C**, non-transgenic exercised (NTG Ex) group; **D**, transgenic overexpressing apoCIII exercised (CIII Ex) group.

**Figure 2:**
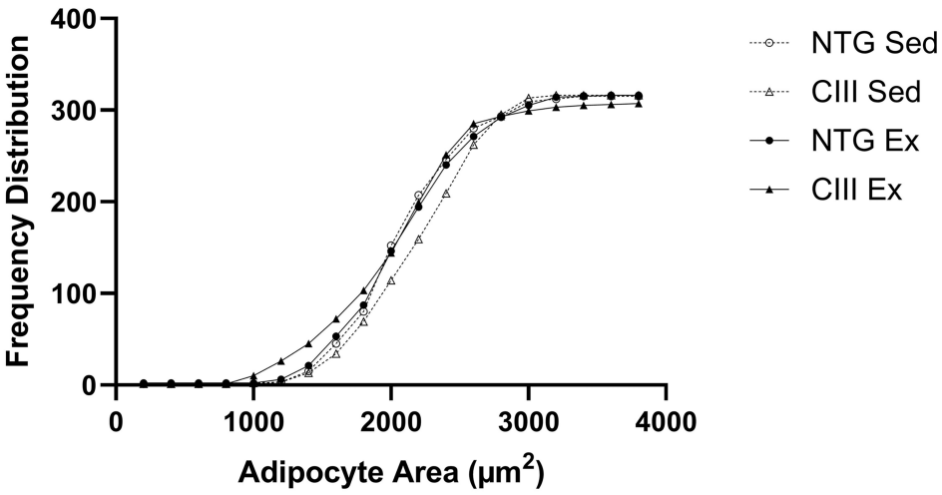
Frequency distribution of adipocyte area. NTG Sed: non-transgenic sedentary group; CIII Sed: transgenic overexpressing apoCIII sedentary group; NTG Ex: non-transgenic exercised group; CIII Ex: transgenic overexpressing apoCIII exercised group.

No significant differences were observed in the body weight, water consumption, liver weight, or liver fat content among the 4 experimental groups (Table 1). ANOVA showed that MICT increased food consumption significantly (P<0.001). Total adipose mass (subcutaneous, perigonadal, retroperitoneal, mesenteric, and interscapular brown adipose tissue) was affected by dyslipidemia, leading to an increase in the CIII groups compared to NTG (P=0.04). The histology of the perigonadal fat (Figure 1) indicated the effect of training in reducing the adipocyte area (Ex *vs* Sed P=0.029). Additionally, the frequency analysis (Figure 2) showed a higher number of small and large adipocytes in the CIII Ex group.

**Table 1:** Body weight, food and water consumption, liver weight, and histology of adipocytes of the study groups.

	Groups				Source of variations		
	NTG Sed	CIII Sed	NTG Ex	CIII Ex	Dyslipidemia	Exercise	Interaction
Body weight (g)	25.9±2.3	26.5±2.1	26.1±0.3	25.2±2.3	ns	ns	ns
Water (mL/w)	31.7±13.7	31.5±3.7	34.2±1.9	35.2±3.2	ns	ns	ns
Food intake (g/w)	21.5±1.7	20.6±1.2	25±2.8^b^	23.7±2.1^b^	ns	<0.001	ns
Total fat weight (%)	3.96±0.94	4.78±1.43^a^	3.34±0.62	4.29±0.87^a^	<0.05	ns	ns
Adipocyte area (μm^2^)	2264±189	2253±409	1935±118^b^	1953±289^b^	ns	<0.05	ns
Liver weight (%)	4.28±0.23	4.5±1.22	4.62±0.1	4.41±0.32	ns	ns	ns
Liver fat (mg/g)	0.028±0.005	0.028±0.008	0.030±0.003	0.041±0.01	ns	ns	ns

Data are reported as means±SD. Statistical analysis by two-way ANOVA, followed by *post hoc* test of Tukey. ^a^Significant effect of apoCIII overexpression (NTG *vs* CIII); ^b^significant effect of exercise (Sed *vs* Ex); ns: not significant. Sed: non-transgenic sedentary group; CIII Sed: transgenic overexpressing apoCIII sedentary group; NTG Ex: non-transgenic exercised group; CIII Ex: transgenic overexpressing apoCIII exercised group.

### Plasma analysis and muscle glycogen

Triglyceride levels in the CIII groups were significantly higher than in the NTG groups (P<0.0001) (Table 2), confirming the overexpression of apoCIII. Exercise reduced plasma triglyceride levels in the CIII Ex group compared to the CIII Sed group (P=0.005). Plasma cholesterol was higher in the CIII Sed group compared to both NTG groups (*vs* NTG Sed P=0.004; *vs* NTG Ex P=0.02), while the CIII Ex group had lower cholesterol levels than its sedentary control (P=0.01). Dyslipidemia had a significant effect in increasing LDL cholesterol levels in the CIII groups (CIII *vs* NTG P=0.005). VLDL cholesterol was significantly higher in the CIII groups (CIII *vs* NTG P=0.0001), and training caused a slight decrease (P=0.03). In addition, exercise increased muscle glycogen stores in trained mice (Sed *vs* Ex P=0.0003).

**Table 2:** Plasma levels of metabolic parameters and muscle glycogen in non-transgenic (NTG) and apoCIII transgenic (CIII) mice after 8 weeks of moderate-intensity continuous training (Ex) or no exercise (Sed).

	Groups				Source of Variations		
	NTG Sed	CIII Sed	NTG Ex	CIII Ex	Dyslipidemia	Exercise	Interaction
TG (mg/dL)	67±14	562±163^a^	61±9	362±58^abc^	<0.0001	<0.05	<0.05
CHOL (mg/dL)	164±44	248±35^a^	173±41^c^	166±57^c^	<0.05	<0.05	<0.05
Glycemia (mg/dL)	87±9	90±12	82±11	96±10	ns	ns	ns
Muscle glycogen (mmol/g)	0.060±0.002	0.060±0.001	0.063±0.001^b^	0.067±0.003^b^	ns	<0.001	<0.05
HDL CHO (mg/dL)	79.7±11.5	81.7±18	76.6±11	85.5±18	ns	ns	ns
LDL CHO (mg/dL)	29.5±18.7	60.6±30.3^a^	24±6.9	61.2±42.3^a^	ns	ns	ns
VLDL CHO (mg/dL)	12.4±1.8	49.7±9.4^a^	9.5±1.6	41.7±6.8^a^	<0.0001	ns	ns

TG: Triglycerides; CHOL: cholesterol; HDL: high-density lipoprotein; LDL: low-density lipoprotein; VLDL: very low-density lipoprotein. Data are reported as means±SD. Statistical analysis by two-way ANOVA, followed by *post hoc* test of Tukey. ^a^Effect of apoCIII overexpression (NTG *vs* CIII); ^b^effect of exercise (Sed *vs* Ex); ^c^ *post hoc* test *vs* CIII Sed; ns: not significant. P values indicate significant effects for dyslipidemia, exercise protocol, and interaction. Sed: non-transgenic sedentary group; CIII Sed: transgenic overexpressing apoCIII sedentary group; NTG Ex: non-transgenic exercised group; CIII Ex: transgenic overexpressing apoCIII exercised group.

### V̇O_2_ kinetics and V̇O_2peak_

**Figure 3:**
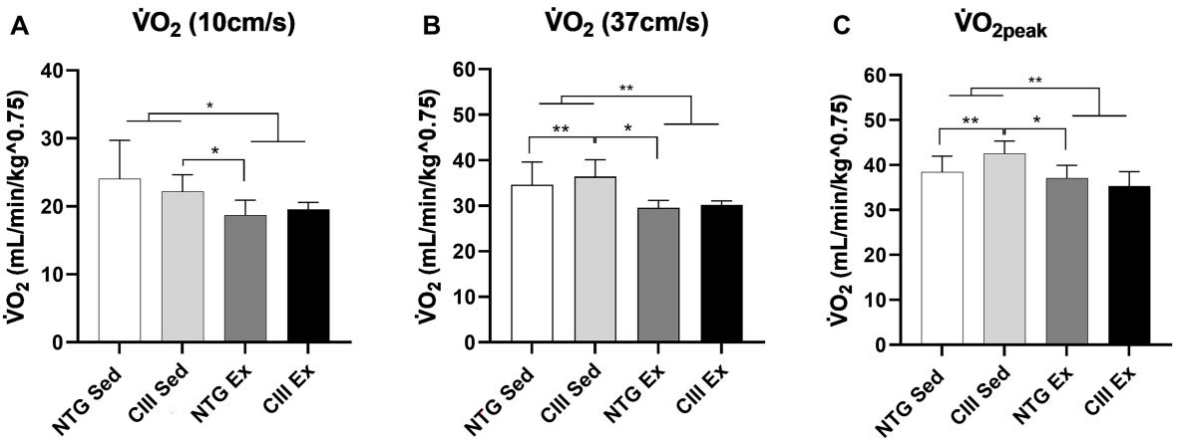
Effect of moderate-intensity continuous training (MICT) on oxygen consumption (V̇O_2_) at 10 and 37 cm/s (**A** and **B**) and V̇O_2peak_ (**C**) of the groups. Sed: non-transgenic sedentary group; CIII Sed: transgenic overexpressing apoCIII sedentary group; NTG Ex: non-transgenic exercised group; CIII Ex: transgenic overexpressing apoCIII exercised group. Data are reported as means±SD. *P<0.05, **P<0.01, two-way ANOVA, followed by *post hoc* test of Tukey.

As an effect of training, trained groups showed lower oxygen consumption at all stages of the effort test (Figure 3). Specifically, in the initial stage of the test, the analysis of variance identified that trained animals had slightly lower oxygen consumption than sedentary animals (P=0.008). The exercise effect increased in the intermediary stages of the test (Figure 3A and B, P=0.0004), persisting until V̇O_2peak_ (Figure 3C, P=0.001).

**Figure 4:**
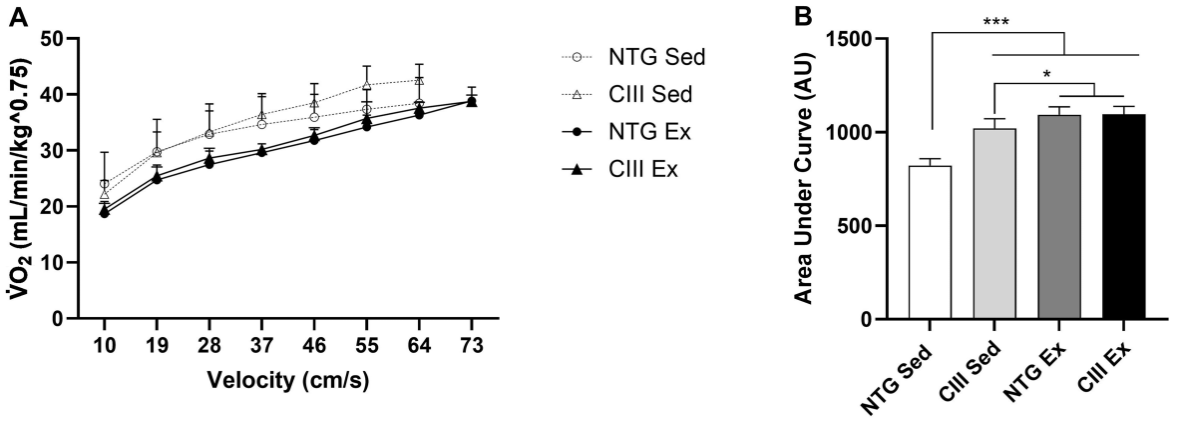
Effect of moderate-intensity continuous training (MICT) on oxygen consumption (V̇O_2_) during the effort test (**A**) and the area under curve for V̇O_2_ of the groups (**B**). Sed: non-transgenic sedentary group; CIII Sed: transgenic overexpressing apoCIII sedentary group; NTG Ex: non-transgenic exercised group; CIII Ex: transgenic overexpressing apoCIII exercised group. Data are reported as means±SD. *P<0.05, ***P<0.0001, two-way ANOVA, followed by *post hoc* test of Tukey.

**Figure 5:**
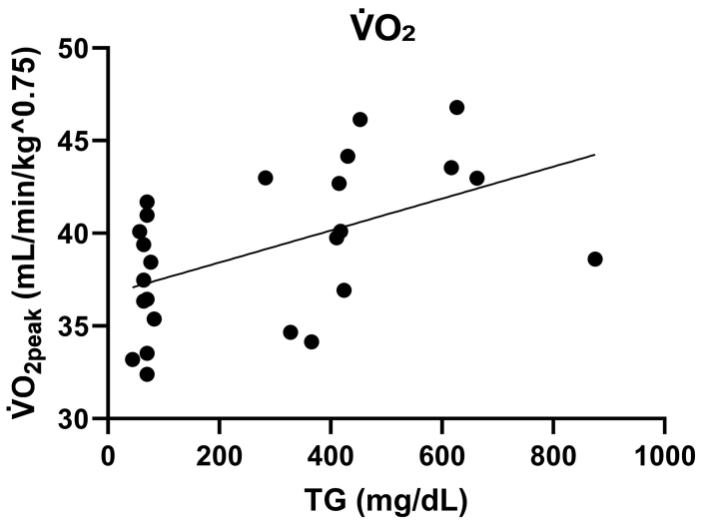
Correlation graph of triglycerides (TG) and peak oxygen consumption (V̇O_2peak_) of groups. Sed: non-transgenic sedentary group; CIII Sed: transgenic overexpressing apoCIII sedentary group; NTG Ex: non-transgenic exercised group; CIII Ex: transgenic overexpressing apoCIII exercised group. r=0.41, P=0.03, Pearson's Correlation.

The kinetics of V̇O_2_ (Figure 4) shows that the trained groups reached higher stages with lower oxygen consumption throughout in the effort test. The area under the curve of V̇O_2_ kinetics shows that both training and dyslipidemia have significant effects in modifying V̇O_2_. The analysis of variance of the area under curve identified that the trained groups had higher oxygen consumption in the effort test (P<0.0001), highlighting the positive effect of training. Additionally, in sedentary animals, dyslipidemia also had an effect on V̇O_2_, showing the same significance for an increase in V̇O_2_ in the CIII groups. This result was also observed through correlation analysis, where we observed a moderate positive correlation between hypertriglyceridemia due to apoCIII overexpression and V̇O_2_ (r=0.4 and P=0.03) (Figure 5). Specifically, for the CIII Sed group, V̇O_2_ in the effort test was significantly higher than its NTG Sed control (P<0.0001).

## Discussion

Dyslipidemia is a highly prevalent multifactorial condition, strongly linked to several chronic non-communicable diseases and is therefore the target of scientific studies aimed at better understanding and dealing with it in different models. This is the first study to investigate the effects of aerobic training on body characterization, feeding behavior, lipid metabolism, glycolytic metabolism, and V̇O_2_ in dyslipidemic animals with human apoCIII overexpression.

Although there were no body weight differences among the experimental groups, the relative mass of adipose tissue in relation to body weight was higher in the CIII animals. The increase in relative fat mass was accompanied by the presence of larger adipocytes, which is consistent with previous data in this animal model obtained from epicardial adipose tissue (24), and MICT reduced adipocyte area. Our data is in agreement with previous studies observing that this type of physical exercise leads to adipose tissue remodeling, reducing adipocyte size without necessarily causing weight loss (25).

The higher plasma triglyceride and cholesterol levels observed in the CIII group compared to the NTG mice reflects the apoCIII overexpression effect of inducing hypertriglyceridemia and elevating cholesterol concentrations (26). The decrease of triglycerides in CIII Ex compared to CIII Sed confirmed our hypothesis that aerobic training can attenuate triglyceridemia in this genetically dyslipidemic animal model. Moreover, the CIII Ex group also exhibited lower plasma cholesterol levels, suggesting that training can reduce cholesterol levels in the presence of apoCIII overexpression, highlighting the atheroprotective effect of exercise.

Previous studies have reported similar findings in dyslipidemic humans, showing that aerobic training can reduce plasma triglycerides and cholesterol levels (27-29). Exercise increases lipoprotein lipase activity and increases triglyceride catabolism (30) with different effects depending on plasma lipid concentration. Interestingly, dyslipidemic individuals are more responsive to exercise training (31).

Studies in dyslipidemic humans undergoing aerobic training have reported similar results to the present study. Circulating apoCIII levels were measured in hypertriglyceridemic individuals before and after 8 weeks of aerobic training, and a decrease in apoCIII levels was found along with a reduction in plasma triglycerides and cholesterol (32).

Studies show that physical training can improve many parameters related to the composition of body fat (33). Different training methods, such as MICT and HIIT (high-intensity interval training), were shown to enhance other markers not shown in our study, such as markers of NAFLD (non-alcoholic fatty liver disease) (34).

One limitation of the present study was that we did not directly assess gene and protein expression of apoCIII, but rather considered the exercise effects in reducing plasma triglycerides and cholesterol. However, it is well-established in the literature that apoCIII concentration is proportional to circulating triglycerides (8). Based on the above, it is possible to suggest that, since exercise resulted in a reduction of circulating triglyceride levels in our animal model, it may also lead to a decrease in apoCIII gene/protein expression.

ApoCIII overexpression was found to increase LDL and VLDL cholesterol in dyslipidemic groups, corroborating the elevated total cholesterol levels. Moreover, the relationship between cholesterol metabolism and exercise is sometimes controversial (31,35). Our study showed a decrease in cholesterol levels with exercise in the CIII Ex group. This is likely due to the increased TG catabolism in beta-oxidation, leading to an increased TG-VLDL degradation and consequently lower LDL production. We emphasize that the modifications induced by physical training on lipid metabolism were evident only in dyslipidemic animals, highlighting the role of exercise in combating dyslipidemia and preserving metabolism in normal subjects (31).

Another important biologic marker of the MICT effects, the skeletal muscle glycogen concentration was increased in the trained groups, with the CIII Ex group presenting higher muscle glycogen compared to the NTG Sed and CIII Sed groups. Muscle glycogen is an important substrate for aerobic exercise, and it is expected that glycogen stores undergo changes as the metabolism adapts to training. Studies also show that a decrease in muscle glycogen content compromises exercise performance (36). Previously, we showed that 4-month-old CIII animals have less glycogen than their NTG controls (18). However, this difference was not found in the sedentary 15-month-old animals, showing that aging in this animal model may also modify their glycogen metabolism.

In addition, the hypertriglyceridemia resulting from the overexpression of apoCIII may have led to a deterioration of the aerobic performance of CIII animals. This was confirmed by the higher oxygen consumption exhibited by sedentary CIII animals when running at the same intensity as their NTG control counterparts. It is noteworthy that CIII animals, characterized by an increased basal metabolic rate due to increased mitochondrial activity (37), may need a higher beta-oxidation and oxidative phosphorylation to achieve the same workload as the NTG group. The fact that dyslipidemia hinders the physical conditioning of dyslipidemic animals from training has been documented in the literature (15). Nevertheless, it is important to note that hypertriglyceridemia is multifactorial, and in our animal model, is caused by a genetic alteration, meaning that this animal, although dyslipidemic, remains mostly healthy.

The CIII Sed group presented higher V̇O_2peak_ than its NTG Sed counterpart at similar effort rate, and even higher than the trained groups, which reached higher stages in the test. Additionally, their V̇O_2_ kinetics had a much larger area under the curve than their NTG Sed control, showing that the CIII Sed animals have higher V̇O_2_ than the NTG Sed group, but a lower one than the trained groups. Contrarily, some studies assessed the V̇O_2_ of hypercholesterolemic animals with apoE and LDL receptor knockout (apoE/LDLR -/-) and found that young transgenic animals had a lower V̇O_2max_ than adult animals, but there were no differences between apoE/LDLR -/- animals and their adult C57Bl6/J controls (38). This effect might be due to the fact that cholesterol, unlike triglycerides, does not participate in metabolic pathways that provide energy for exercise training.

As an effect of training, we identified an increase in V̇O_2_ in both exercised groups. The trained groups performed better in the effort test, as they covered greater distances, reached higher speeds, and had a lower relative V̇O_2_ during the different stages of the test, indicating a lower energy demand than the sedentary animals. There was no significant difference between the CIII and NTG exercised groups, indicating that training improved the metabolism of dyslipidemic animals similarly to the NTG group.

### Conclusion

In summary, MICT was able to reduce triglyceridemia and cholesterol levels in CIII mice, demonstrating that training can modify the metabolic profile of dyslipidemic animals, even through genetic induction. Additionally, training decreased adipose area, increased muscle glycogen concentration, and improved oxygen consumption, showing the effectiveness of the proposed moderate-intensity and long-duration protocol. Even though the overexpression of apoCIII has been identified as a crucial factor in the pathogenesis of dyslipidemia, MICT has emerged as an effective tool to normalize the glycemic and lipid profile, as well as hypertensive factors in individuals with dyslipidemia, potentially preventing atherosclerosis. MICT is a low-risk and easily applicable exercise protocol, being considered a promising option to reverse the comorbidities associated with dyslipidemia and promote cardiovascular health.

### Limitations

Following the adherence criteria to the training protocol, animals that did not complete at least 80% of the training protocol were excluded from the final analysis. Due to training adversities, the sample size was reduced from 10 animals to 5 in the CIII Ex group and 6 in the NTG Ex group.
